# On the accuracy of sequence methods for baroreflex sensitivity estimation

**DOI:** 10.1007/s13246-023-01380-y

**Published:** 2024-04-02

**Authors:** Hasana Bagnall-Hare, Violeta I. McLoone, John V. Ringwood

**Affiliations:** 1 Department of Electronic Engineering, Maynooth University, Maynooth, Co. Kildare Ireland; 2 Department of Aero, Mechanical and Electronic Engineering, South East Technological University, Waterford, Co. Carlow Ireland

**Keywords:** Sequence methods, Baroreflex sensitivity, System identification

## Abstract

In the absence of a true gold standard for non-invasive baroreflex sensitivity estimation, it is difficult to quantify the accuracy of the variety of techniques used. A popular family of methods, usually entitled ‘sequence methods’ involves the extraction of (apparently) correlated sequences from blood pressure and RR-interval data and the subsequent fitting of a regression line to the data. This paper discusses the accuracy of sequence methods from a system identification perspective, using both data generated from a known mathematical model and spontaneous baroreflex data. It is shown that sequence methods can introduce significant bias in the baroreflex sensitivity estimate, even when great care is taken in sequence selection.

## Introduction

The baroreflex sensitivity (BRS) quantifies the relationship between a change in blood pressure and a change in R-R interval (the time elapsed between two successive R-waves of the QRS signal on the electrocardiogram), where R-R interval variations are considered a response to changes in blood pressure (BP). A high BRS is indicative of a strong relationship between BP and R-Rinterval, therefore demonstrating a robust baroreflex, with the opposite suggesting the baroreflex is impaired. Analysis carried out in [1] showed that a high BRS is linked to vagal (or parasympathetic) effects and not BP buffering (controlled by peripheral resistance), which explains why BRS assessment has been shown to provide insight into the probability of cardiovascular events, especially in patients with a number of underlying conditions [2]. The current gold standard for BRS estimation, the Oxford method [3], is invasive and can be unsafe to perform on patients at risk of cardiovascular events, typically those patients that may benefit most from continuous BRS monitoring. In an attempt to find an accurate non-invasive BRS estimation method, which can be performed in a clinical setting, a number of non-invasive methods have been developed using spontaneous data [4]. Spontaneous data, in this case, is considered spontaneous fluctuations in BP and HR, recorded while the subject is in standing and supine positions, without any external stimulus. The non-invasive methods developed range from spectral analysis methods [5] to autoregressive methods [6], and everything in between [7– 9], with sequence methods emerging as a popular method [10, 11].

Results obtained from recent non-invasive BRS methods are being compared to those obtained using the sequence methods [12– 14], identifying the sequence methods as the *de facto* gold standard for non-invasive BRS estimation. As sequence methods become more prevalent, their reliability as accurate BRS estimators is questioned. In 2019, *Silvia et al* [15] investigated the BRS estimates obtained using sequence methods applied to the complete BP and R-R interval series, in comparison to those obtained from a low-pass (LP) and high-pass (HP) filtered series. The results show correlation between estimates obtained from the original series and the HP series, with little to no association with the LP estimates. This suggests that sequence methods only quantify short term fluctuations in the baroreflex, such as respiratory fluctuations, failing to accurately capture long term effects, like sympathetic mediated changes in BP and HR. In 2020, *Wessel et al* [16] concluded that the sequence selection criteria are incapable of distinguishing between the random and baroreflex controlled sequences, their results suggesting that sequence methods have“a potentially large methodological bias as an estimate for the baroreflex sensitivity”. In 2020, *Wessel et al* [16] concluded that the sequence selection criteria are incapable of distinguishing between the random and baroreflex controlled sequences, their results suggesting that sequence methods have“a potentially large methodological bias as an estimate for the baroreflex sensitivity”. In 2020, *Chen et al* [13] calculated BRS in rats using the traditional, invasive, Oxford method and compared them to estimates obtained using the sequence methods (applied to spontaneous readings). Their results show a positive bias in the mean gain estimates, and a high standard deviation, for the BRS estimates obtained using sequence methods compared to those obtained using the Oxford method. The high standard deviation suggests that, while the gains are higher than the Oxford method, the estimates are not just offset by a constant across all cases, but that the bias introduced varies from case to case. This disparity between cases makes it difficult to account for the bias when trying to assess if a patient has an impaired or non impaired baroreflex when using sequence methods for BRS estimation.

The main contribution of this paper is a critical examination of the theoretical foundation behind the use of sequence method for baroreflex sensitivity estimation. The analysis aims to explain the persistent positive bias seen in BRS estimates obtained using sequence methods when compared to alternative non invasive BRS estimation methods [4, 13, 16]. While other studies show a positive bias in BRS estimates obtained using sequence methods, this paper determines the source of the bias in the BRS estimates, and if refinements made to the sequence selection process are sufficient to overcome the bias.

It should be noted that the analysis in this paper primarily focusses on the‘beat-to-beat’baroreflex, mediated primarily by A-fibers, characterized by myelinated axons with a lower activation threshold (approximately 60 mmHg) and near-constant activity throughout the cardiac cycle, encoding blood pressure levels through a frequency-modulated code of action potentials. A-fibers also feature a high conduction speed, relative to C-fibers, which are typically only activated during noxious or perilous stimuli, such as substantial increases in blood pressure, which may be characteristic of the Oxford method [17–19]. As a result, the results in this paper, and results from other non-invasive BRS studies, may not be directly comparable to those obtained using the invasive Oxford method for BRS estimation.

The remainder of the paper is organised as follows. Sections 2.2 and 2.3 describe the state of the art of BRS estimation and the development of sequence methods; Sect. 2.4 analyses the effect sequencing data has on gain estimation; Sect. 2.5 details the development of a controlled numerical example where sequence methods can be tested on a system with a known gain; Sect. 2.6 demonstrates the prevalence of positive gain bias in BRS estimates using sequence methods, in comparison to methods from the control systems sciences; Sect. 3 presents the results of the paper; Sect. 4 discusses the results of the paper; and Sect. 5 draws the conclusions on the study.

## Materials and methods

### The baroreflex

**Fig. 1:**
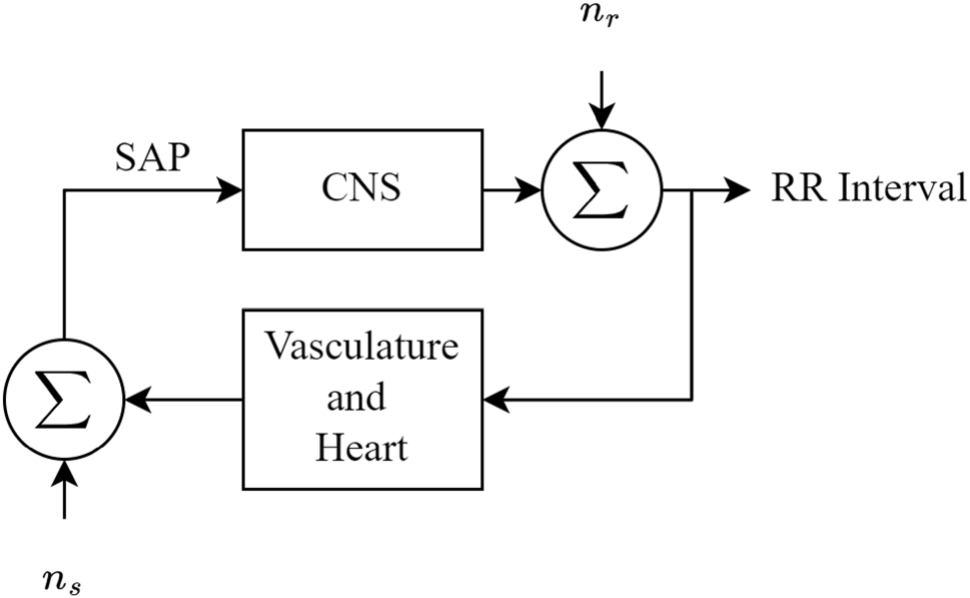
The baroreflex loop with two external noise sources, $n_r$ representing respiration and $n_s$ representing central modulation of the sinus node

The baroreflex is the homeostatic mechanism that maintains BP, by correcting small changes in BP, detected through baroreceptors, via alteration of cardiac output and peripheral resistance. Figure 1 depicts the baroreflex loop as a feedback control loop, which regulates RR interval via the central nervous system (CNS). In this instance, both respiration and modulation of the sinus node are considered noise sources. The gain of the CNS block represents BRS. It is also worth noting that the effectors of BP operate on a variety of time scales. Spectral indices around 0.25Hz typically represent respiration, oscillations around 1Hz represent cardiac activity, with 0.1Hz reflecting sympathetic and vagal tone [1, 20].

### BRS estimation

The Oxford method [3] is the current gold standard for BRS estimation, using a vasodilator or constrictor to elicit a strong change in BP, and the subsequent RR-interval change recorded. The relationship between BP and RR-interval is plotted, a linear regression line [21] is fitted to the data, and the regression coefficient (slope) identified as the BRS gain. Alternatively, researchers [22] have suggested employing mathematical functions with a nonlinear sigmoidal-like shape to represent the baroreflex buffering response to the pharmaceutical stimuli observed. The approach in [22] aims to further improve the accuracy of the estimated BRS gain. The invasive nature of the test makes it impossible to carry out in a routine clinical setting, expensive, and can be unsafe to perform on patients with a number of underlying conditions [23]. Since the ability to accurately, and safely, assess BRS is most beneficial for patients with a history of cardiac events or cardiac related underlying conditions, the majority of which cannot safely undergo the Oxford procedure, the need for a simple, non-invasive alternative is paramount. To date, no gold standard for non-invasive BRS estimation exists, though a number of methods have been proposed, ranging from spectral analysis methods to regression methods, including sequence methods [4].

The majority of non-invasive BRS estimation methods developed can be categorised as time- or frequency-domain approaches. Frequency domain approaches typically investigate the BP and RR interval variability through spectral anaylsis [4]. Frequency domain approaches rely on the assumption that the frequency range of interest is the region representing heart rate variability under the influence of the baroreflex. If the frequency range is correct, in theory the effects of noise sources in the BRS estimate are mitigated. While time domain methods focus on the identification of changes in RR interval driven by changes in BP, and quantifying the strength of the response, time domain methods attempt to mitigate the noise sources through the selection of baroreflex driven portions of the recordings.

### Sequence methods for BRS estimation

**Fig. 2:**
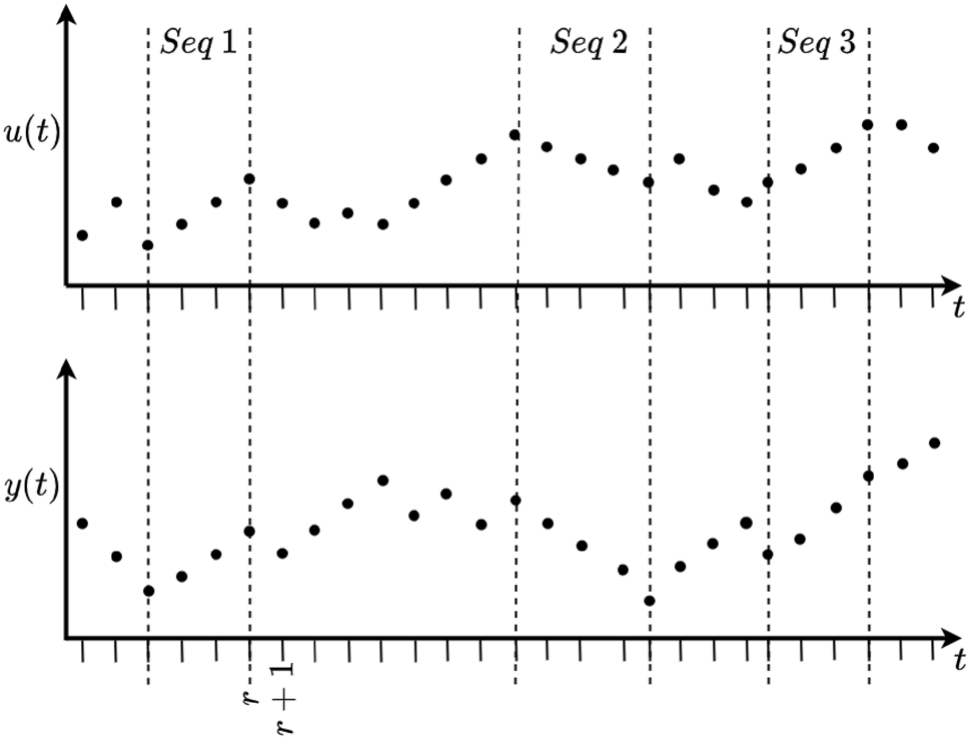
Example of valid sequence selection. The graph highlights three instances where three, or more, matching increases/decreases in both the input (*u*(*t*)) and the output (*y*(*t*)) are observed

Over the past three decades, sequence methods have become a popular method for non-invasive measurement of BRS [10, 24–28]. A sequence method, first introduced by Di Rienzo in 1985 [29], analyses BP and ECG signals, recorded without any external stimulus, for correlated increases or decreases in both the input (BP) and output (RR-interval). Under the sequence method philosophy, a set of data points is considered a valid sequence if it has three, or more, consecutive increasing or decreasing points in input and output data. Examples of positive-going and negative-going sequences are shown in Figure 2. Following sequence selection, a regression line is fit to each individual input/output sequence. The ‘gain’ for the sequence is the regression coefficient and the BRS for the data set is determined as the average of all the regression coefficients.

In contrast, Gouveia *et al* [27] fit a global regression line through all selected sequences, a method that was further developed in [30] and [31].

In 1988, *Bertinieri et al* [32] further refined the sequence selection criteria to include a minimum changes of at least $\ge 1mmHg$ in blood pressure, $\ge 4 ms$ in RR-interval, and a delay of one beat is assumed [32]. Since then, various researchers have chosen to use a variety of minimum thresholds, delays and some include a minimum correlation coefficient (*r*) in an attempt to make the gain estimates more robust to noise. In 2009, *Laude et al* [33] investigated the optimal combination of minimum sequence lengths, variable thresholds, delays and *r* values, in an attempt to standardise the sequence selection criteria. The authors carried out their experiment on mice data sets, investigating BRS estimation using sequences of various lengths, using various BP/R-R interval thresholds and different delays between the input and output. It also investigates whether a minimum *r* is required. The study concluded that sequence methods, when applied to data from mice, should consider sequences of three beats, or more, with a delay of zero or three beats. Thresholds for the *r*, SBP, or PI were deemed unnecessary.

### Analytical overview of sequence methods

The increase in popularity of sequence methods calls for a rigorous analysis of the sequence methods to verify their validity as a reliable identification method. In this section, the effects sequencing data has on any associated noise contamination and, in turn, it’s effects on gain estimation, are considered.

#### Sequences within a system identification framework

Spontaneous BP and ECG readings, used for non-invasive BRS estimation methods, such as the EuroBaVar dataset [4], are continuous BP and ECG readings taken from individuals without any external interventions/stimulus imposed for the purpose of the recordings i.e. the closed-loop baroreflex is intact. When dealing with closed-loop data, a number of fundamental properties must be considered when modelling the system’s behaviour.

Identification methods, used to elicit open-loop dynamics from closed loop data, can be divided [34] into three categories: (a) direct methods, where the measured input–output signals are used and the feedback loop is ignored, (b) indirect methods, where some information about the feedback is available and an effective open-loop input signal can be formed, and (c) joint input–output methods, where both input and output signals are treated as outputs driven by noise and the feedback system. Spontaneous BP and ECG readings do not provide any information about the feedback loop or noise present in the system, meaning that direct methods are appropriate when dealing with the development of BRS estimation methods from spontaneous BP and ECG readings. However,it should be noted that direct methods, applied as if the feedback does not exist, only work well if the true system can be described within the chosen model structure (both the dynamic model and the noise model). In time-domain identification, given that sampled data is used, a discrete time system representation is used, via a general time-domain model structure of:

1 $$\begin{aligned} y(t) = G(q)u(t) + H(q)e(t) \end{aligned}$$

where *y*(*t*) is the output signal (RR-interval), *u*(*t*) is the input signal (BP), and *e*(*t*) is an unknown white noise sequence, with a mean value of zero and assumed to be uncorrelated with past or future values of *u*(*t*). *t* is the discrete-time index. *G*(*q*) is the open-loop system of interest, with *H*(*q*) representing the noise model.

If an inadequate noise model (*H*(*q*)) is used, deviations from the true noise characteristics will introduce bias in the estimate of system *G*(*q*). Sequence methods consider $G(q) = k$, where *k* is a positive rational number, and $H(q) =0$. Since sequence methods, in essence, consider the system to be a simple gain and do not account for any dynamics, in either the main system or the noise model, a bias in the BRS estimate is highly likely.

#### Generic analysis of sequence methods

Suppose that the input signal for a system (such as the baroreflex) is described by a ramp of the form:

2 $$\begin{aligned} u(t) = p \; t \end{aligned}$$

where *t* is the discrete-time index and *p* can take on the values $+1,-1$. This input is passed through the system described by a simple gain, with the output corrupted by measurement noise $e(t) = [-n, n]$, as:

3 $$\begin{aligned} y(t) = s k u(t) + e(t) \end{aligned}$$

where $k < n$. We assume that the gain, *k*, is +ve, though *s* can take on the values $+1,-1$. In the case of baroreflex sensitivity, *s* is always +1 (i.e. a +ve change in blood pressure causes a +ve change in RR-interval), but we will retain the flexibility in order to determine general conclusions. Now, a sequence is selected where the output is going in a consistent direction, in a similar manner to ‘Seq 1’ shown in Figure 2,

4 $$\begin{aligned} Y(r,i) = [y(r-i) \; y(r-i+1) \; \ldots \; y(r-1) \; y(r)] \end{aligned}$$

and we assume that the next point, $y(r+1)$, is inflected compared to the points in *Y*(*r*, *i*). Four cases, corresponding to all possible combinations of *p* and *s* will now be considered.

*Case 1* $p = +1\;, \; s = +1$ The input to the system is a +ve going ramp:

5 $$\begin{aligned} u(t) = t \end{aligned}$$

The system is described by:

6 $$\begin{aligned} y(t) = k \;u(t) \; + \; e(t) \end{aligned}$$

For an inflection:

7 $$\begin{aligned} y(r+1)+e(r+1) < y(r)+e(r) \end{aligned}$$

But

8 $$\begin{aligned} y(r+1) = y(r)+ k \end{aligned}$$

giving, from (7), that

9 $$\begin{aligned} e(r+1) < e(r) - k \end{aligned}$$

and *e*(*r*) is positively biased with respect to $e(r+1)$, with the result that the estimate for *k* from the sequence *Y*(*r*, *i*) is positively biased.

*Case 2* $p = +1\;, \; s = -1$ The input to the system is a +ve going ramp:

10 $$\begin{aligned} u(t) = t \end{aligned}$$

The system is described by:

11 $$\begin{aligned} y(t) = -k \;u(t) \; + \; e(t) \end{aligned}$$

For an inflection:

12 $$\begin{aligned} y(r+1)+e(r+1) > y(r)+e(r) \end{aligned}$$

But

13 $$\begin{aligned} y(r+1) = y(r)- k \end{aligned}$$

giving, from (12), that

14 $$\begin{aligned} e(r+1) > e(r) + k \end{aligned}$$

and *e*(*r*) is negatively biased with respect to $e(r+1)$, with the result that the estimate for *k* from the sequence *Y*(*r*, *i*) is negatively biased.

*Case 3* $p = -1\;, \; s = +1$ The input to the system is a -ve going ramp:

15 $$\begin{aligned} u(t) = -t \end{aligned}$$

The system is described by:

16 $$\begin{aligned} y(t) = k \;u(t) \; + \; e(t) \end{aligned}$$

For an inflection:

17 $$\begin{aligned} y(r+1)+e(r+1) > y(r)+e(r) \end{aligned}$$

But

18 $$\begin{aligned} y(r+1) = y(r)- k \end{aligned}$$

giving, from (17), that

19 $$\begin{aligned} e(r+1) > e(r) + k \end{aligned}$$

and *e*(*r*) is negatively biased with respect to $e(r+1)$, with the result that the estimate for *k* from the sequence *Y*(*r*, *i*) is negatively biased.

*Case 4* $p = -1\;, \; s = -1$ The input to the system is a -ve going ramp:

20 $$\begin{aligned} u(t) = -t \end{aligned}$$

The system is described by:

21 $$\begin{aligned} y(t) = -k \;u(t) \; + \; e(t) \end{aligned}$$

For an inflection:

22 $$\begin{aligned} y(r+1)+e(r+1) < y(r)+e(r) \end{aligned}$$

But

23 $$\begin{aligned} y(r+1) = y(r)+ k \end{aligned}$$

giving, from (7), that

24 $$\begin{aligned} e(r+1) < e(r) - k \end{aligned}$$

and *e*(*r*) is positively biased with respect to $e(r+1)$, with the result that the estimate for *k* from the sequence *Y*(*r*, *i*) is positively biased.

### Artificial numerical example

**Fig. 3:**
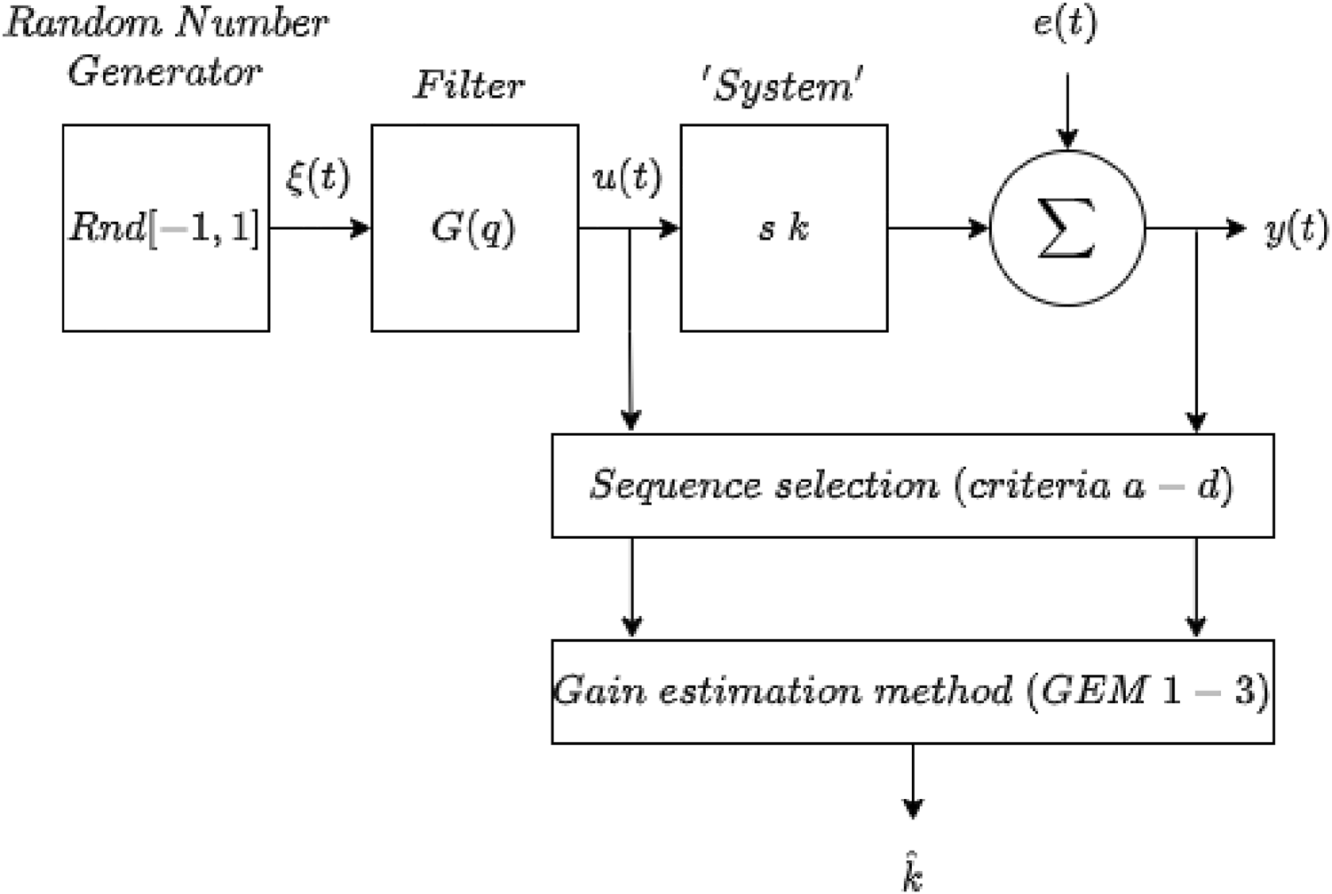
Numerical experiment set up. The input signal *u*(*t*) is formed by passing a sequence of random numbers through a low pass filter. The system is described by a simplain gain, in this case 2. The output *y*(*t*) is formed by the output of the filter and additive white noise *e*(*t*). Sequence selection is determined by the valid sequence criteria (outlined in Table 2). From the sequences the system’s gain is estimated using a gain estimation method (outlined in section 2.5.3)

Section 2.4.2 provides a generic analysis of sequences and highlights potential limitations associated with using sequence methods as a method of gain estimation, this section builds on that by investigating these points in the context of a controlled numeric example. In this section, an ideal system is simulated to examine, experimentally, the effect of sequence methods under known, controlled, conditions. Specifically, cases 1 and 3, detailed in Sect. 2.4.2, where the system is a simple positive gain, with additive noise, with both positive- and negative-going input sequences, depicted in Figure 3, are examined within a numerical framework.

#### Problem setup

##### Input signal

 The input signal, a surrogate for BP, is generated using a zero mean, random number generator, which is passed through a discrete time $7^{th}$ order Butterworth filter with transfer function:

25 $$\begin{aligned} F(q) = \frac{b_o+b_1q + ... + b_nq^n}{1+a_1q+ ... + a_nq^n} \end{aligned}$$

where *n* is the order of the model, *q* is the backward shift operator and the $a_i$ and $b_i$ are chosen to achieve unity dc gain. The signal was filtered both forward and backwards, giving a zero phase distortion, and an effective filter order of 14. Note that there is a strong relationship between $\Omega _c$, the normalised filter cut-off frequency, and the maximum sequence length $L_{max}$ that can be achieved in *u*(*t*), given by the approximate relation:

26 $$\begin{aligned} L_{max} \approx 3.26 + 7 e^{-8 \Omega _c} \end{aligned}$$

**Fig. 4:**
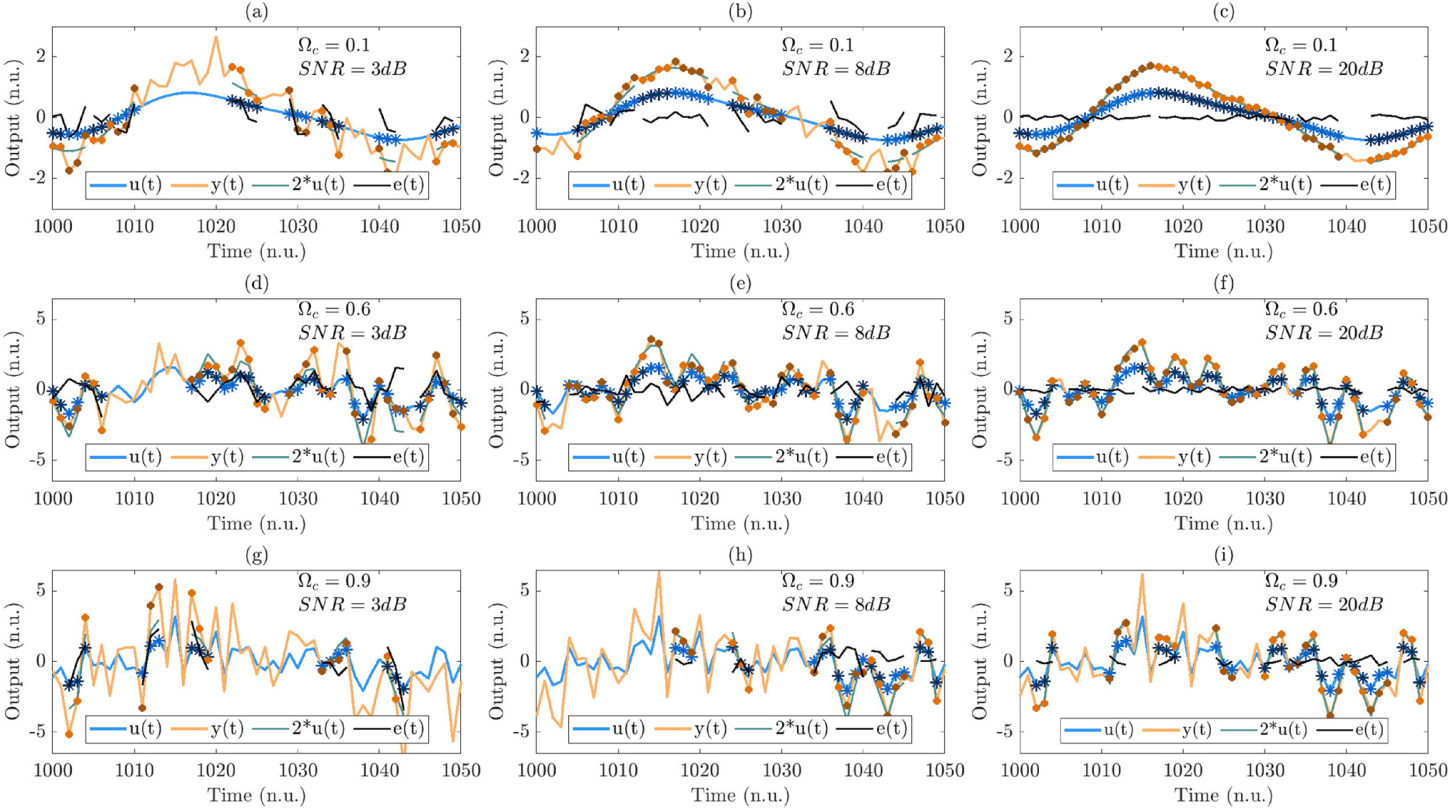
Examining the effect of $\Omega _C$ and *SNR*, on the maximum sequence length achieved in *u*(*t*) and *y*(*t*).  denotes the input signal *u*(*t*) with  and  marking alternate valid sequences,  denoted the output of the system before the noise signal is added ($2^*u(t)$),  denoted the noise signal *e*(*t*) and  denotes the output signal *y*(*t*) with  and  marking alternate valid sequences

However, the max sequence length, determined by the simultaneous minimum in both *u*(*t*) and *y*(*t*), is also dependent on the signal-to-noise ratio (SNR) in *y*(*t*), which is determined by the amplitude, *m*, of the measurement noise, *e*(*t*). The relationship between $\Omega _c$ and the maximum sequence length that can be achieved in *u*(*t*), and *SNR* and the maximum sequence length that can be achieved in *y*(*t*) is illustrated in Figure 4. The average sequence length for various combinations of $\Omega _c$ and *SNR* is shown in Table 1.

**Table 1:**
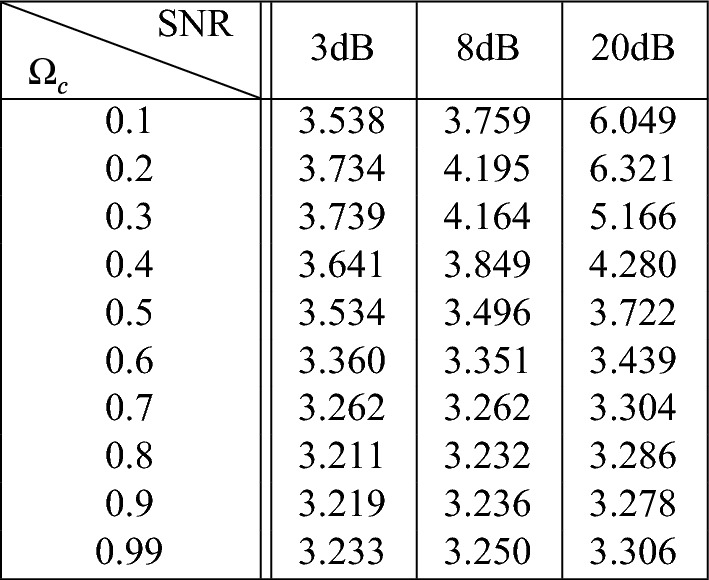
The average sequence length for various combinations of $\Omega _c$ and SNR

##### System

 The system, a surrogate for baroreflex sensitivity, is a simple gain of 2, with additive zero mean, white noise. The noise signal is a surrogate for unquantified factors that influence the baroreflex.

##### Output signal

 The output of the simulated system, a surrogate for R-R interval, is:

27 $$\begin{aligned} y(t) = 2u(t) + e(t) \end{aligned}$$

where *u*(*t*) is the input, *e*(*t*) is zero mean white noise and *y*(*t*) is the output.

#### Sequence selection

After generating both *u*(*t*) and *y*(*t*), valid sequences are extracted. In the literature, a number of variations on the criteria for a valid sequence are presented, as detailed in Sect. 2.3, this study considers four sets of selection criteria, as documented in Table 2.

**Table 2:** Sequence selection criteria used for the numerical example

Criteria	Sequence length	*r*	Input threshold	Output threshold
a	3+	0	No	No
b	3+	0.85	No	No
c	3+	0.7	Yes	Yes
d	4+	0	Yes	Yes

The input and output thresholds used for criteria c and d in Table 2 are calculated using:

28 $$\begin{aligned} Input \ threshold = \dfrac{BP \ threshold}{\sigma _{BP}^2} \times \sigma _u^2, \end{aligned}$$

where $\sigma _{BP}^2$ denotes the variance in BP, $\sigma _{u}^2$ denoted the variance on the input signal, and

29 $$\begin{aligned} Output \ threshold = \dfrac{RR \ threshold}{\sigma _{RR}^2} \times \sigma _y^2, \end{aligned}$$

where $\sigma _{RR}^2$ denotes the variance in RR-interval and $\sigma _{y}^2$ denote the variance on the output signal. The relationship between $\sigma _u^2$ and $f_c$ is characterised as:

30 $$\begin{aligned} \sigma _u^2 = f_c \sigma _{rnd}^2 \end{aligned}$$

where, $\sigma _{rnd}^2$ is the variance on the signal generated by the random number generator. The relationship between $\sigma _y^2$, $f_c$ and SNR is characterised as:

31 $$\begin{aligned} \sigma _y^2 = k_1 f_{c} e^{-k_2 SNR} + k_3f_{c} \end{aligned}$$

The input and output variances change with $\Omega _c$ and SNR, in turn varying the input and output thresholds in each case; $\sigma _{x}^2$ decreases as $\Omega _c$ decreases, while $\sigma _{y}^2$ decreases as $\Omega _c$ decreases and increases as SNR decreases.

These four criteria were chosen to examine the effects of sequence selection refinements and to what extent, if any, they improve the accuracy of the gain estimates, in comparison to the original sequence method. Criteria set a represents the original sequence method [29]; Criteria set b, a sequence method with an additional threshold on *r*; Criteria set c, a method with both *r* and minimum signal thresholds; and Criteria set 4, a method with a longer minimum sequence length and minimum signal thresholds. It should also be noted that while, in the literature, some methods account for a delay between the input and output, a lag is not considered in the current numerical case as there is no delay in the system generating *y*(*t*) from *u*(*t*).

#### Gain estimation methods

To examine the accuracy of sequence methods (GEM 2 and GEM 3) as a gain estimation method, their performance is compared to a sequence-free gain estimation method, GEM 1. GEM 1 through 3 are defined as follows:

*Gain estimation method 1 (GEM 1)* Linear regression applied to the complete data set, where the gain is the regression coefficient [21].

*Gain estimation method 2 (GEM 2)* Linear regression applied to all data points identified as valid sequences, where the gain is the regression coefficient [27].

*Gain estimation method 3 (GEM 3)* Linear regression applied to individual (valid) sequences, where the gain is the average of all the individual regression coefficients [35].

**Fig. 5:**
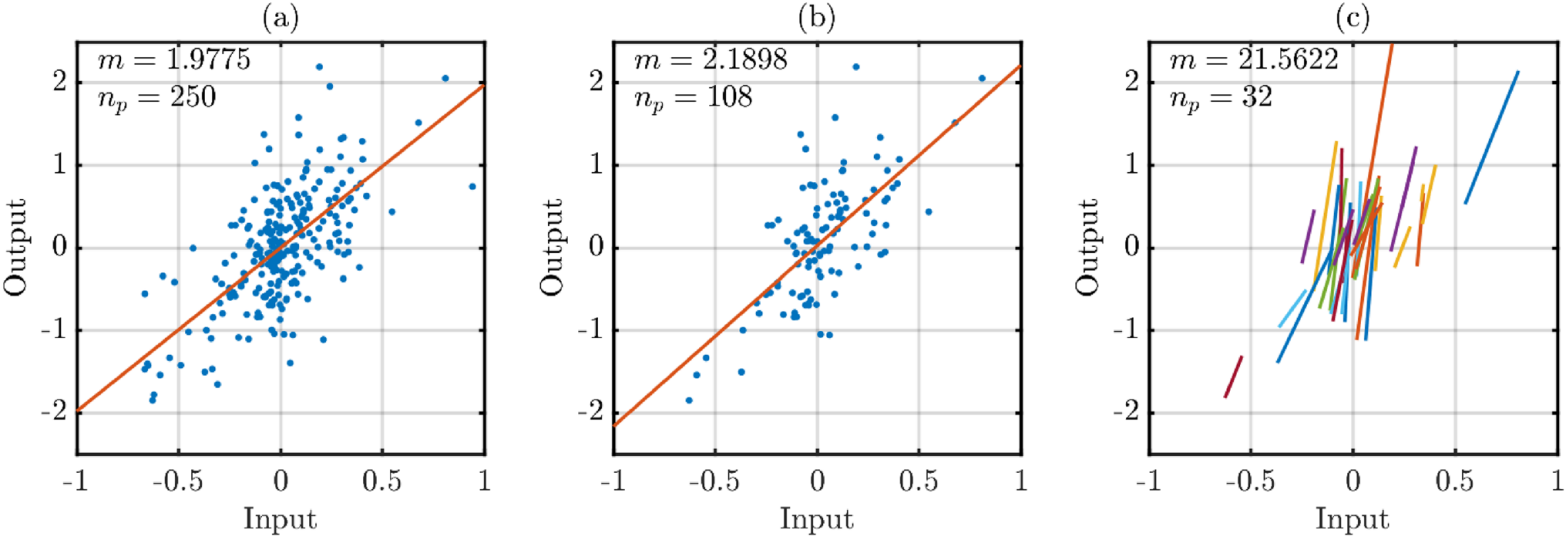
Three estimation methods applied to the numerical example: **a** GEM 1: a linear regression fit to the entire data sets, **b** GEM 2: a linear regression fit to selected (sequence) data points, and **c** GEM 3: a linear regression fit to each individual sequence. m represents the overall gain estimate from each method, $n_p$ represents the number of data points considered in (**a**) and (**b**), while $n_{seq}$ represents the number of sequences considered in (**c**). For this illustration, a specific cut-off frequency ($\Omega _c= 0.1$), SNR ($= 0dB$), and a reduced number of data points in *u*(*t*) and *y*(*t*), are chosen to clearly highlight the differences between the three methods

Methods 2 and 3 are applied four times, using the various sequence selection criteria detailed in Table 2. Figure 5 provides an illustration of the three gain estimation methods in action, highlighting the differences in their use of the data, and showing that GEM 2 results in a smaller number of data points compared to GEM 1, with multiple (individual) regression lines for GEM 3.

### Experimental analysis using the EuroBaVar data set

The efficacy of sequence methods, as a BRS estimation method, is now examined, via the EuroBaVar experimental data set [4]. BRS estimates, obtained from the spontaneous blood pressure signals, are examined for trends that align with those seen in the numerical example, i.e. positive bias in BRS estimates calculated using the sequence methods, compared to alternative, system identification (SI) based methods.

#### EuroBaVar data set

The EuroBaVar dataset [4] is an open source dataset consisting of 42 files corresponding to spontaneous, non-invasive BP and HR readings from 21 subjects in both supine and standing positions for approximately 10 min. The data is recorded on a beat by beat basis, using a Finapres®system, for BP, and an ECG system, for RR-interval. Subjects 13 and 18 are considered to have an impaired baroreflex, while a number of subjects are considered to be at risk of impaired baroreflex due to underlying conditions. Valid, non-invasive, BRS estimation methods must be capable of clearly distinguishing subjects 13 and 18 as impaired, while also (ideally) highlighting the subjects at risk of an impaired baroreflex with a lower BRS estimate than the healthy volunteers.

#### BRS estimation methods employed

To examine the accuracy of sequence methods as a BRS estimation method, BRS estimates calculated using 7 estimation methods applied to the EuroBaVar dataset are compared. Methods 1a through 1d, outlined in Table 3 represent 4 sequence methods with equivalence to the sequence selection criteria used in the numerical case, outlined in Table 2.

Methods 2a through 2c represent BRS estimation methods based on a rigorous SI approach developed by *McLoone et al* [36]. This method was selected for comparison due to its utilisation of ARMAX models, which account for a dynamic noise component, unlike sequence methods. In addition, in [36] a weighting function applied to the frequency spectrum places greater importance on frequencies associated with the baroreflex, thereby further reducing the effect of noise on the BRS estimate. The data preprocessing, and the initial steps, are the same for each of methods 2a $\rightarrow$ 2c, as follows:

- Resample signals *u*(*t*) and *y*(*t*) at 1.5*Hz*.
- Pass *u*(*t*) and *y*(*t*) through Butterworth filters with high pass $\Omega _c=0.05$ and low pass $\Omega _c=0.5Hz$ cut-off frequencies. The frequency range used is that specifically associated with the human baroreflex.
- An autoregressive moving average (ARMAX) model is fit to the data using a prediction error method.
- A Gaussian-based non-uniform weighting function *W*(*f*) is applied to the frequency response of the identified model, where
  32 $$\begin{aligned} W(f) = e^{-\frac{(f-f_0)^2}{2 \sigma ^2}}, \end{aligned}$$
  with $\sigma$ a constant, which determines the width of the Gaussian weighting function.
- In general, a representative average value on a continuous frequency variable *f*, over a range $[f_{min},f_{max}]$, can be obtained as:
  33 $$\begin{aligned} BRS(f_{min},f_{max}) = \frac{1}{N_w} \int _{f_{min}}^{f_{max}} W(f) . |G_{yu}(f)| df, \end{aligned}$$
  where *W*(*f*) defines the ‘weighting’ applied at each frequency over the interval $[f_{min},f_{max}]$, $f_0$ is the centre frequency of each band, and $N_w$ is a normalisation factor, where
  34 $$\begin{aligned} N_w = \int _{f_{min}}^{f_{max}} W(f) df. \end{aligned}$$
  If $G_{yu}$ is only available at $N_f$ discrete frequency points $f_i = f_{min} + i \; \Delta f \;\;, \;\; i = 0,1,\ldots ,N_f-1$, where $\Delta f$ is the frequency interval between points, then equation (33) becomes:
  35 $$\begin{aligned} BRS = 1/N_w \sum _{i=0}^{N_f-1} W(i) . |G_{yu}(i)|, \end{aligned}$$
  with $$N_w = \sum _{i=1}^{N_f}W(i)$$.

*Method 2a*

- BRS gain is calculated using equation (35) with a frequency range 0.05 to 0.15*Hz*

*Method 2b*

- BRS gain is calculated using equation (35) with a frequency range 0.15 to 0.4*Hz*

*Method 2c*

- BRS gain is the average of the gain estimates obtained from methods 2a and 2b.

**Table 3:** Valid sequence criteria used with the experimental data

Criteria	Sequence length	*r*	BP threshold (mmHg)	RR threshold(ms)
a	3+	0	0	0
b	3+	0.85	0	0
c	3+	0.7	4	1
d	4+	0	4	1

## Results

### Numerical example results

The analysis carried out in Sect. 2.4.2 demonstrates the effect of sequencing data sets on the noise properties of the system, demonstrating that the gain obtained from the sequenced data is positively biased. The numerical example investigates these findings through a dedicated numerical example, where the gain $=2$. Table 4 shows the gain estimates obtained using the gain estimation methods outlined in Sect. 2.5 (GEM 1, 2 & 3). Over the years, a number of refinements to the sequence selection criteria (Sect. 2.3) proposed, to improve the fidelity of sequence methods and reduce the effects of noise on the gain estimates. To investigate the effects of the refinements on the gain estimates, four different sequence criteria (outlined in Table 2) were applied to the numerical data.

Considering the original sequence method (GEM 3 using criteria a in Table 3), it is apparent that this sequence method returns higher gain estimates than those obtained using linear regression analysis (GEM 1), with the accuracy of the estimates decreasing as the amount of noise added to the system increases. While GEM 2 does provide a significantly better estimate compared to GEM 3, using all the data (via GEM 1) proves to be the most resilient to noise.

The effect of the refinements on the gain estimate were investigated through the addition of threshold and correlation coefficients limits to the sequence selection process (criteria b and c). The refinements do improve the accuracy of the gain estimates, but only marginally. However, the increase in minimum sequence length from 3 to 4 (criteria d) provides a more significant improvement in accuracy of GEM 3 across a number of cases, though fidelity still suffers for cases with significant additive noise.

**Table 4:**
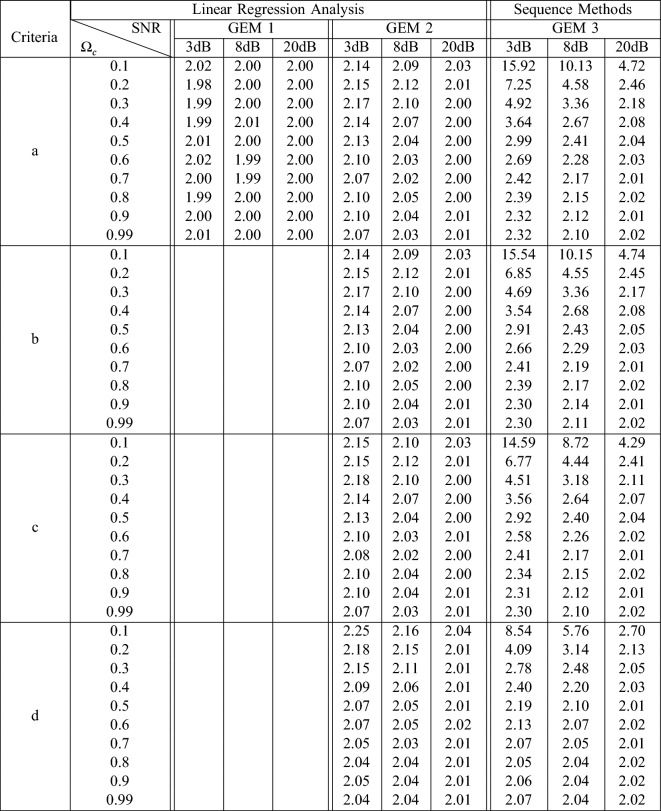
Gain estimation using three methods, (i) GEM 1: linear regression applied to entire data set, (ii) GEM 2: linear regression applied to data points selected through sequence methods and (iii) GEM 3: average slope of the regression coefficient of each sequence

The criteria column corresponds to the valid sequence criteria outlined in Table 2, $\Omega _c$ represents the normalised cut-off frequency of the LPF and *SNR* represents the SNR of the *e*(*t*).It should be noted that the true gain of the system is 2

### EuroBaVar data set results

Considering the analytical examination (section 2.4.2), and the increased gain estimates observed when using the sequence methods in the numerical example, the results suggest that consistently higher BRS gains are obtained using sequence methods. From Table 5, which contains the BRS estimates obtained using the methods outlined in Sect. 2.6, the results show that, in the majority of cases, the BRS estimates obtained using sequence methods are consistently higher than those obtained using a SI-based approach that accounts for the closed-loop nature of the baroreflex, with the exceptions highlighted by a yellow cell colour.

Table 5 also identifies those subjects deemed to have an impaired baroreflex, i.e. subject 13 and 18, highlighted by a green row colour, *and* the patients with underlying conditions with risk of an impaired baroreflex, highlighted by a blue row colour. For these special cases, it should be noted that not only do the sequence methods overestimate the gain, but there are also a number of cases where a BRS estimate cannot be obtained from the data using sequence methods, the majority of which are subjects deemed to have an impaired baroreflex. This means that sequence methods frequently fail in cases where accurate BRS estimation is vital.

**Table 5:**
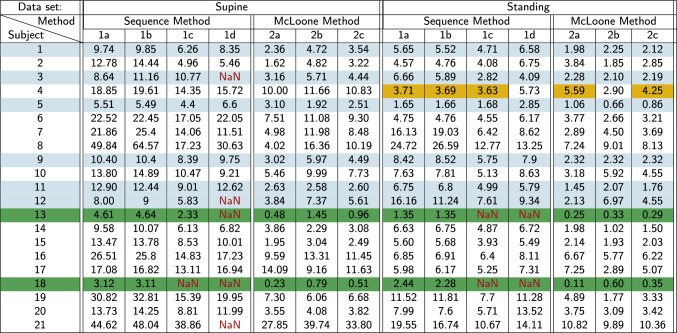
BRS estimates from 6 sequence methods applied to the 21 participants of the EuroBaVar study

(*1a*)Sequence method: 3, or more, consecutive paralleled increases/decreases in both BP and RR, a minimum ${{r}}$ of 0.7, a minimum BP threshold of 1mmHg and a minimum R-R interval threshold of 4ms, (*1b*)Sequence method: 3, or more, consecutive paralleled increases/decreases in both BP and R-R and a minimum ${{r}}$ of 0.7, (*1c*)Sequence method: 4, or more, consecutive paralleled increases/decreases in both BP and RR, a minimum BP threshold of 1mmHg and a minimum R-R threshold of 5ms, (*2a*) LF estimation from McLoone's method, (*2b*) HF estimation from McLoone's method, and (*2c*) average of the LF and HF estimations from McLoone’s method

The green highlighted rows indicate the two patients with impaired baroreflexes, with the blue highlighted rows indicate patients with underlying conditions that may have a lowered BRS estiamtion than the healthy volunteers. The yellow highlighted cells indicate the cases where sequence method estimates were not higher than SI based estimates. NaN indicates an inability to obtain a BRS estimate

## Discussion

### Numerical example

Sequencing data for the purpose of BRS estimation is considered to help reduce the effects of unmeasurable external stimuli on the R-R and BP signals by choosing ‘baroreceptor-like’ events, with the objective of increasing the accuracy of the gain estimates obtained [29]. From Table 4, in the case of the numerical example where the noise introduced into the system is exactly known and specifiable, it is apparent that the sequence methods overestimate the gain of the system, with particular sensitivity to measurement noise. In comparison the two linear regression methods both perform better where significant noise is present; however, it should be noted that the case where only sequenced data points (GEM 2) are used, the gain estimated is still biased. This indicates that the data points not selected contain important information about the system and are useful for improvement in gain estimate fidelity.

While more recent sequence methods contain refinements to the sequence selection criteria, involving the use of correlation coefficient limits and input/output thresholds, the results in Table 4 show that they provide little improvement, in the majority of cases. In the cases that there is improvement, the difference, unfortunately, is marginal.

### Experimental example

Following on from Sect. 4.1, BRS estimates obtained using sequence methods (see 2.6.2 Method 1a–d) are compared with those obtained using a rigorous SI protocol (see 2.6.2 Method 2a–c) for instances of gain overestimation, similar to that seen in the numerical example. From Table 5, it is apparent that the BRS estimates obtained using the sequence methods have consistently higher values than those obtained with the SI based identification method.

Concerns regarding overestimation evident in BRS estimates obtained using sequence methods, does not arise from the presence of overestimation, or even the extent of overestimation. Rather, the inconsistency in the degree of overestimation between subjects is a cause for concern. If overestimation observed in BRS estimates is consistent between cases, it might be manageable by adjusting the estimates (or the threshold for low BRS) to account for the gain error. However, from Table 5, the disparity between estimates obtained using the sequence and the McLoone methods vary from case to case, suggesting that the noise present in the recordings vary from subject to subject. This is expected, as the metabolic demands of other subsystems in the body vary from person to person, at any given time. The inter-person variability would translate to the BRS estimate under the guise of noise in methods that do not account for a noise model, such as sequence methods.

However, one main cause for concern with sequence methods is the inability to detect sufficient valid sequences to make a realistic BRS estimate in patients with an impaired baroreflex. Sequence methods require concurrent increases/decreases in BP and RR-interval, which are not always present in subjects with impaired baroreflex, as seen in patients 13 and 18, in Table 5.

### Observations and future work

This study investigates the apparent overestimation of BRS gain observed when using sequence methods in a number of studies [4, 13, 16], by analysing the effect sequencing data has on gain estimation (Sect. 2.4.2). The analysis suggests that the sequence methods are limited by the fact that sequenced R-R interval data contains a significant amount of noise (ie. non-baroreflex related effects). The events excluded by the sequence methods appear to contain key information about the gain of the system, with exclusion of the corresponding data points leading to persistent overestimation of the gain of the system.

The spontaneous nature of the R-R interval and BP readings could play a role in the inability of sequence methods to distinguish between ‘baroreflex like’and‘un-baroreflex’events. The Oxford method applies linear regression analysis to a dataset where the observed change in R-Rinterval is primarily driven by a sudden strong change in BP, minimising the effect of non-baroreflex effectors on R-R interval. The inclusion of planned movements that are known to stimulate the baroreflex [37] during the recordings could potentially improve the accuracy of sequence methods by ensuring the sequences selected are ‘baroreflex like’. Known baroreflex stimulating movements include the Valsalva maneuver [38], timed sit-to-stand maneuver [39], and the neck chamber technique [40], all of which would continue to allow for BRS estimation in a routine, clinical setting. The recording and analysis of experimental data, where patients carry out the above movements, would allow researchers to investigate if the addition of dedicated baroreflex stimulating movements improve the accuracy of the sequence methods.

## Conclusion

The work presented in this paper provides a detailed analysis of sequence methods and their efficacy as a BRS estimation method, from a SI perspective. An initial analytical approach outlines how sequencing data sets can lead to biased gain estimates. This is then confirmed through a numerical example, demonstrating that, in the case of a simple gain system, the sequencing of data leads to gain overestimation, especially in cases where there is significant noise present. Recent refinements made to sequence methods, viz. the addition of a correlation metric and a minimum threshold improve the situation to some extent, but are insufficient in completely rectifying the bias in the gain estimates. The limitations of sequence methods outlined from the analytical study, and the numerical example, are borne out in the analysis of the experimental data. This can be seen in the consistently higher BRS estimates obtained using sequence methods, in comparison to those obtained using a rigorous SI method. The analysis carried out, especially in the experimental analysis, shows potential cause for concern with using sequence methods as a BRS estimation method, and subsequently the estimates being used as a diagnostic tool for subjects with an impaired baroreflex.
